# A National Virtual Peer Support Group for Women Veterans Living with Breast Cancer: Lessons from the Field

**DOI:** 10.3390/ijerph23060817

**Published:** 2026-06-19

**Authors:** Jenny K. Cohen, Kara Zamora-Rogoski, Caitlin L. McLean, Mariam E. Jacob, Evana Mack, Haley Moss, Aimee Kroll-Desrosiers

**Affiliations:** 1 San Francisco Veterans Affairs Health Care System, Division of General Internal Medicine, San Francisco, CA 94121, USA; kara.zamora@va.gov (K.Z.-R.); evana.mack@va.gov (E.M.); 2 Department of Medicine, University of California San Francisco, San Francisco, CA 94143, USA; 3 Northern California Institute for Research and Education (NCIRE), San Francisco, CA 94121, USA; 4 Veterans Affairs Sierra Nevada Health Care System, Reno, NV 89502, USA; caitlin.mclean@va.gov; 5 Durham Veterans Affairs Health Care System, Durham, NC 27705, USA; haley.moss1@va.gov; 6 Duke Cancer Institute, Durham, NC 27701, USA; 7 Department of Health Promotion and Policy, University of Massachusetts Amherst, Amherst, MA 01003, USA; akroll@umass.edu; 8 Center for Healthcare Delivery Science, Beth Israel Deaconess Medical Center, Boston, MA 02215, USA

**Keywords:** peer support, qualitative research, veteran

## Abstract

**Highlights:**

**Public health relevance—How does this work relate to a public health issue?**

- Cancer is one of the top three divers of morbidity and mortality in the US and as such presents a major public health threat that not only impacts the health and wellbeing of millions of Americans each year but also poses a financial and psychosocial threat. Peer support groups are a low-cost potent intervention that has been shown to improve psychosocial wellbeing for individuals going through shared experiences such as cancer. Our work builds the case that for women veterans with breast cancer, virtual peer support groups are a powerful tool for improving this population’s wellbeing.

**Public health significance—Why is this work of significance to public health?**

- Peer support is an important aspect of cancer care. Our work illuminates how a virtual platform can be leveraged across a national catchment to offer a low-cost intervention to a geographically disparate and heterogenous group of veterans going through a similar experience to build community, decrease social isolation, and improve wellbeing.

**Public health implications—What are the key implications or messages for practitioners, policy makers and/or researchers in public health?**

- Apply lessons learned from the creation of the first ever national VA support group for women veterans with breast cancer to an analogous cohort of patients who may be distributed across wide geographic swaths and experiencing similar clinical and psychosocial challenges.
- Identify the necessary resources and methodologies to develop and implement peer-facilitated cancer support groups for other unique populations of patients undergoing cancer treatment or survivorship surveillance.

**Abstract:**

Within the Veteran’s Health Administration (VHA), peer support specialists (PSSs) have traditionally worked in mental health and behavioral health settings. PSS-facilitated cancer support groups are less common and underused in this setting. The purpose of this study was to understand the acceptability, feasibility, and perceived benefits of a PSS-facilitated peer support group for women veterans with breast cancer. Semi-structured interviews were conducted among veteran participants and health system leaders (HSLs) and were recorded, transcribed, and analyzed using rapid qualitative analysis. Key findings from interviews with veterans and HSLs emerged across several domains: The value of shared experiences, peer status and “matching”, virtual aspect, group structure, beneficial topics, and desired outcomes. Veteran participants greatly valued the ability to share experiences and connect with other women veterans with breast cancer and shared a desire for the facilitator to be a peer with an overlapping shared lived experience as they described benefits from peer interactions including supportive coping and instrumental coping. Veterans also reflected on the acceptability of the group being virtual, and uncovered attitudes and preferences regarding group structure, beneficial topics, and desired outcomes. HSLs noted that target outcomes might be linked to feeling connected with community and having an increased feeling of support. Findings suggest that cancer support groups, unlike more traditional mental health support groups at the VHA, may require greater specificity regarding programmatic content and PSS-cancer-experience-matching for group experience to feel authentic and meaningful.

## 1. Introduction

Cancer is one of the top three drivers of morbidity and mortality in the US and, as such, presents a major public health threat that impacts the health and wellbeing of millions of Americans each year. Peer support groups are a potent intervention that have been shown to improve psychosocial wellbeing for individuals going through shared experiences such as cancer [1]. Geographic heterogeneity and small incidence of cancer among women veterans relative to male veterans enrolled in Veterans Health Administration (VHA) results in a disproportionate reliance on community care services for women with cancer. This is particularly true for women veterans with breast cancer [2]. Prior studies show that community care utilization itself is associated with increased psychosocial burden and distress [2,3,4,5,6,7]. Previous research found that women veterans with cancer reported feeling alienated, isolated, and anxious around care coordination [8,9]. Additionally, women veterans expressed a desire to talk to other women veterans going through similar experiences. Those previously published concerns suggest that there is an urgent need for specialized sex-sensitive, veteran-centered, high-quality, and accessible specialty care and support to improve psychosocial outcomes for women veterans with cancer [3,4,5,6,7,10].

Support groups have been shown to improve psychosocial wellbeing for individuals going through a shared experience, such as living with cancer [11]. While definitions of support groups vary in the health sciences literature, one definition of support groups are unstructured gatherings that have minimal facilitation, rely on support arising organically from participants, and can be facilitated by professionals, community members, or peers [12]. The VHA has a rich history of offering support groups to veterans living with behavioral and mental health challenges. To strengthen their ability to offer these groups, VHA created a network of peer support specialists (PSSs) who serve as paraprofessionals within the “consumer–providers” model and facilitate support groups as well as engage with veterans one-on-one [13]. PSSs are veterans with shared lived experiences as those with whom they work to provide guidance and to foster a sense of belonging through their work as one-on-one coaches and support group facilitators [3,4,14]. Up until recently, PSSs have been utilized in the mental health space providing group and individual support for veterans with mental health and/or substance use related needs. The VHA initiated their first peer support program in 2005; as of 2026 there are 1280 peer support specialists nationwide, of which 269 are women [5]. All PSSs are trained in group facilitation, system-navigation, and coping skills [5].

While VHA has a long track record of offering peer support groups in the mental health space, cancer support groups are less common, and PSS-facilitated cancer support groups are novel. Prior studies show that PSS-facilitated groups have the potential to improve female veterans’ wellbeing [15]. Considering that women veterans, particularly rural-dwelling, with cancer report feeling isolated and worried about their diagnosis, treatment, and care coordination [2,3,16], we developed and piloted a novel PSS-facilitated support group with the objective of helping women veterans with breast cancer with particular attention to potential relevance for rural veterans. In this demonstration project, using the Plan-Do-Study-Act framework, we conducted a formative evaluation using qualitative methodology, understanding the acceptability, feasibility, and impacts of implementing and promoting peer support groups for this unique cohort of veterans.

## 2. Research Strategy and Materials

While the number of women veterans is increasing in the U.S., women veterans with cancer live in geographically diffuse areas. To address this, we partnered with the VA National Tele Oncology’s (NTO’s) Breast and Gynecologic Oncology System of Excellence (BGSOE) to develop and pilot a national virtual peer support group. For group recruitment, BGSOE created a health informatics tool (VetConnect), which identifies veterans with breast and gynecological cancers, including those whose care is delivered by both VA and community providers, by using a combination of ICD-10 codes, and claims data that is identifiable through a Power BI dashboard. This dashboard contains individual-level demographic information and basic diagnostic information that is used operationally by BGSOE for veteran outreach; study staff accessed this only due to approval under VA policies. In the fall of 2023, the BGSOE Nurse Navigator used the dashboard to identify and call veterans with a new diagnosis of breast cancer in the fall of 2023. The Nurse Navigator was tasked with asking standardized intake questions with the goal of offering support group enrollment to all veterans who were called (September 2023–October 2024). Between 1 September 2023 and 31 October 2024, the BGSOE RN Navigator spoke to 302 women veterans with new diagnoses of breast cancer. Of the veterans who were successfully contacted, 51 expressed interest in participating in the virtual support group and were referred to the support group, 98 were not asked, and 152 declined referral to the support group. Veterans who expressed interest in the support group were scheduled and the PSS initiated a 1:1 introductory call (see Table 1 for participant demographics) and those who chose to participate in the group were scheduled into six one-hour weekly support group virtual visits. In total, 41 unique veterans participated in the support group between September 2023 and October 2024. Using urban communing areas (RUCA) and Federal Office of Rural Health Policy (FORHP) definitions, we attributed rurality codes to support group participants (a score of 1 is a metropolitan area and 10 indicates a rural area) [17].

Support groups were hosted virtually using the VHA’s Virtual Care (VVC) platform, the platform used for telehealth visits throughout VHA. Assistance was provided for veterans having VVC connectivity issues. Groups were held at 5 p.m. EST weekly and led by a female veteran PSS with a history of non-gynecologic/breast cancer, as we were initially unable to identify a PSS with specifically a history of breast cancer, and approached this as a limitation to feasibility.

The PSS group facilitator was an existing full-time VHA employee. As part of routine job duties, she was already trained as a PSS, including in support facilitation, clinical documentation, and leading support groups in the mental health space. She was able to incorporate the support group and associated administrative responsibilities into her existing PSS role.

Support groups consisted of weekly one-hour-long sessions and were grouped into six-week “waves”. New veterans were allowed to join at the start of each wave, and veterans were invited to participate in as many waves as they desired. Veterans were expected to attend all sessions in the six-week wave.

### 2.1. Peer Support Group

**Figure 1:**
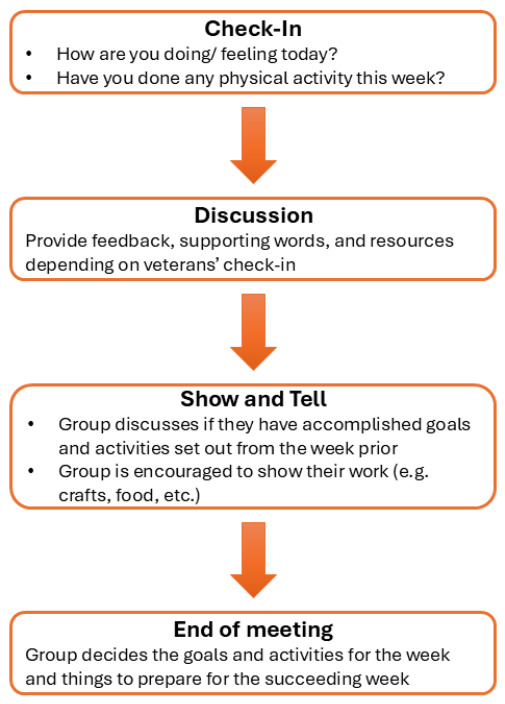
Support group framework.

Since this was the first support group of its kind, we identified best practices for cancer support group structure and curriculum through consultation with the VA Peer Support Community of Practice, VHA Mental Health guidelines, and with subject matter experts at three unique VA facilities. Every group started with a check-in, followed by a wellness-focused activity, with the remainder of the session dedicated to a theme chosen the week before by a group member (Figure 1). At the end of each session, the PSS assigned wellness-focused activities (e.g., healthy eating, gardening, or crafting) and in subsequent weeks group members would come to the session and share what they practiced.

### 2.2. Feasibility Evaluation

We developed original semi-structured interview guides for veteran participants and HSLs (see Supplemental Material). The veteran interview guide focused on uncovering factors that prompted them to join the group; views on the virtual platform, session structure, session topics, and number of sessions; and comfort in sharing in the group, and suggestions for improvement. The HSL interview guide focused on elucidating views on the objective of the group, meeting patient needs, desired patient outcomes, implementation challenges, factors that would support program sustainability, and suggestions for improvement, as well as feedback heard from participants. Verbal informed consent was obtained from each participant prior to an interview. Veteran support group participants were recruited to participate in a qualitative interview through a convenience sampling approach. Veterans who had completed at least one six-week cycle of the support group were informed by the PSS that they would be invited by a team member to be interviewed after their last session to discuss their experiences in the group. HSLs were invited to participate in an interview by email. The qualitative team consisted of one medical anthropologist and health services researcher (KZ) and one clinical psychologist and women’s health researcher (CM) trained in qualitative research methods. Interviews were conducted by qualitative team members via telephone (veterans) or a Microsoft Teams meeting (HSLs). Interviews lasted approximately 30 min. Veteran interviews were audio recorded for analysis and HSL interviews were transcribed in real time via Microsoft Teams. Teams auto-transcribed transcripts were reviewed for accuracy by the interviewers. All recorded audio data was stored on secure VA servers. All data will be retained in accordance with local VA policy and audio files will be destroyed after the completion of the project. Recruitment tracking spreadsheets were stored in secure MS Teams folders (as approved by VHA) and study IDs were not linked to identifiable information.

We used a rapid qualitative analysis method designed for health services research which allows for qualitative results to be analyzed concurrently with data collection to inform the development and evaluation of interventions and implementation strategies [18,19]. Numeric study identifiers were assigned to each participant at the time of the interview. Guided by our semi-structured interview guides, we created summary templates using Microsoft Excel organized by topical areas for each stakeholder group. A qualitative team member listened to each audio-recorded interview and populated a summary template with key points and quotations. To ensure reliability and mitigate researcher bias, a second qualitative researcher listened to the audio-recorded interview and reviewed the summary for accuracy. To synthesize data, we used Matrix Analysis, an approach to displaying data to highlight commonalities and differences and to identify patterns and relationships [18,19,20]. We created veteran and HSL findings matrices using Microsoft Word-organized topical areas from the interview guides to compare feedback across participants. A qualitative team member (KZ) took the lead on reviewing and consolidating aggregated data within topical areas of each matrix to identify emergent patterns within and across domains. This process, known as data reduction, is used to organize, focus, and highlight material so conclusions can be drawn from the data [21]. We paired findings with direct quotations to ensure alignment with participants’ voices. A second qualitative researcher (CM) reviewed and provided feedback on the preliminary findings. The qualitative team met bi-weekly to reach consensus, preliminary findings were discussed with the larger study team at monthly meetings, and agreement was reached through an iterative process.

This demonstration project used a Plan-Do-Study-Act framework for process improvement, which is an iterative process used to inform group changes and not an end state. Thus, thematic saturation was not seen as an end goal as iterative changes will continue to be made over additional group cycles. Instead, information power estimation is a model that accounts for five areas that affect sample size: (1) the aim of the study (broad vs. narrow), (2) sample specificity, (3) use of established theory, (4) quality of dialogue, and (5) analysis strategy. The qualitative aim was relatively narrow and specific, had moderate sample specificity (women veterans with breast cancer diagnosis from diverse backgrounds), and used applied theory rather than exploring new theory. We expected the quality of dialogue to be high as the questions were specific to the PSS group and structured categorical responses informed the rapid qualitative analysis approach. Within this framework, study participants provided adequate information to inform necessary adaptations and advance the project to the next stage of inquiry [22].

In addition to qualitative interviews, feasibility was assessed descriptively through recruitment and delivery metrics, including the number of recruitment calls completed, number of waves delivered, staffing integration within existing PSS roles, and ability to deliver sessions using the VVC platform.

## 3. Results

**Figure 2:**
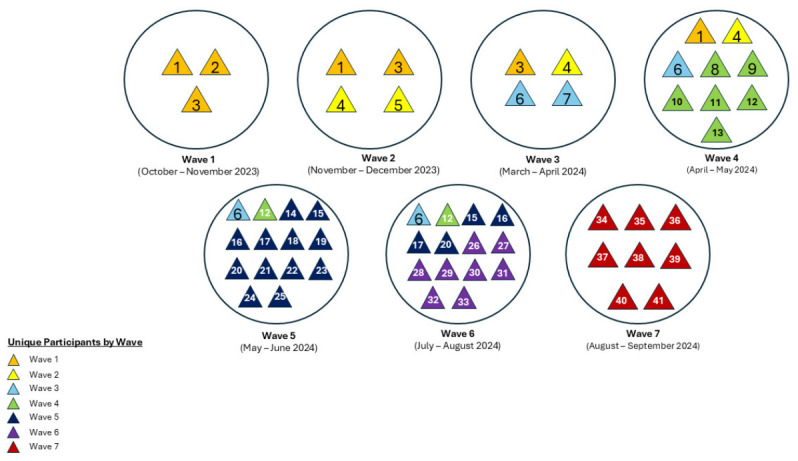
Visualization of support group participation throughout the first seven waves of support groups from October 2023–September 2024. Each triangle represents a unique veteran. The color of the triangle indicates the first wave they participated in and the number within the triangle represents a unique veteran.

The pilot included seven waves of six-week virtual peer support groups delivered between October 2023 and September 2024, with a total of 41 unique participants, *n* = 9 (22%) who participated in more than one six-week wave. Figure 2 shows a visual representation of unique vs. repeat participants who went through the first seven waves of the support group. Each triangle represents a unique veteran and the color corresponds to the wave in which they started. The first six-week wave included three participants. Wave 2 included two veterans who participated in the first wave plus an additional two new participants. A similar pattern played out for Wave 3, and Wave 4 saw representation from Wave 1, 2, 3 as well as six new participants. Wave 5 was the first wave that lacked representation from Waves 1 or 2, and Wave 7 was unique in that it contained all novel participants (Figure 2). Of note, we had hoped to oversample rural residing veterans; however, we were unable to and thus, only 6 of the 41 veterans were rural (Table 1).

Eleven veterans and three HSLs completed qualitative interviews after the conclusion of the pilot period. Key findings from interviews emerged across several domains: the value of shared experiences, peer status and “matching”, virtual aspect, group structure, beneficial topics, and desired outcomes. In the quotations presented, participant identifiers are coded for confidentiality: veteran participants are labeled as V001–V011, and HSL as H001–H003.

### 3.1. The Value of Shared Experiences

Veteran participants reported looking forward to discussing shared experiences in the virtual peer support group. As one participant stated, “at the time I was going back into a depression, so for me to have a group of peers that I could talk with and listen to, and focus on something other than myself, know that I wasn’t the only one struggling through things, was very helpful” (V010). V010 noted that she would recommend the group to a friend because having shared experiences makes you feel supported versus alone, stating:

“*Yes I would, because you get to learn… things that you’re experiencing, you’re not the only one experiencing them, you have the support of people going through, who have been through, who are about to go through, the same things that you’re going through with breast cancer. I’ve been cancer-free for 10–11 years, but it still hurts, it’s still something that affects me every day*.”

Veterans also valued hearing about, and being able to learn from, others’ experiences. V009 described the value of joining the group soon after she was diagnosed with breast cancer, stating:

“*I was still very much in the, ‘wow, this just happened and I don’t even know what this means for me’. So it was, well, let me let me hear other people’s experiences and see if there’s something there that, you know, maybe it’ll trigger something that I might need help with that I just don’t even realize that I need help with that. So, that’s what initially [made me] willing to give [the group] a shot.*”

V010 relatedly shared when it comes to discussing tough decisions that can come with cancer treatment, “It’s easier to talk to people who are going through it than to talk to somebody who’s never gone through it and never had to make those choices… they can’t relate to what you’re talking about.” Another veteran, V004, shared that she felt stressed pre-surgery and that it was helpful to hear others’ perspectives on what surgery might entail, stating “How is this gonna look? What’s the healing time? And everybody’s healing rate is different… going to the [virtual group] sessions they were kinda telling me what their experiences were so it was ‘okay, maybe it’s not so bad’ kind of thing.”

Reflecting on the potential benefit of the virtual peer support group, one HSL participant, H001, described how the group can provide a space for support and sharing knowledge. H001 explained, “I think having a cancer diagnosis can be really isolating, and I don’t think people want to really talk about their experiences. And so I think the benefit is having a meeting [with] a mediator [who] prompts discussion. I think people maybe feel a little bit more comfortable with a prompt… I just think there’s so much value of talking to others that have gone through something, to know whether or not what you’re feeling is normal or not normal or whether or not you should have concerns about your treatment or not.” H001 added to her view that patients may also feel intimidated to ask certain questions of their healthcare providers and might feel more comfortable sharing knowledge and experiences with peers.

Several veteran participants also described looking forward to and enjoying connecting with other females who served in the military because of the shared understanding of the military and the VA. V005 explained, “I liked that we were all veterans. It’s hard to connect to my other [community-based breast cancer support] group because I am the only veteran. And there are things obviously that we all have experienced or a language we all speak that civilians do not understand.” Similarly, V003 shared, “I think a big part of it was people get to vent and say things to people that understand them. I think that was one of the more important parts. A lot of us veterans don’t have a safe space, and I thought that was really great.” Participants added that being able to discuss experiences of navigating VHA-covered community-based services was paramount, with one participant stating, “Because at that time I… hadn’t had radiation yet, and so I was able to—along with the other veterans—share my experience and how to get appointments… and so, all of us, we was able to learn how to call, and who to call, to get appointments set up, whether it be in the community or at the VA and that was very beneficial” (V011).

Relatedly, HSL participant H001 spoke to the challenges that veterans can face needing to move between VHA and community-based care. H001 concluded that this can result in fragmented care for the veteran, even more so among veterans living in rural communities where fewer healthcare options are available, stating:

“*For breast cancer, a third of their patients get care within the VA, a third of the patients get care all in the community, and a third get this kind of combination of both where they get some of their care within the VA and some of them in the community. So it’s really, you know, quite fragmented*.”

HSL participant H003 similarly echoed veterans’ views on the value of speaking with peers who have shared experiences navigating VHA care, stating:

“*Peer support is instrumental in that cancer journey, if you will, having folks that have navigated VA care… most of our patients get care in the community, so having the ability to talk to people who have navigated part of their [care in] the VA and then part of it being in the community and handling and figuring those sorts of things out.*”

(H003)

### 3.2. Importance of Peer Status and “Cancer Type Matching”

Overall, veteran participants described the veteran PSS facilitator to be kind, welcoming, personable, easy to talk to, and open to suggestions. At the same time, two veteran participants reported feeling surprised that the PSS facilitator was a survivor of a non-breast or gynecologic cancer, rather than breast cancer. V009 shared that she was under the impression that everyone was going to have breast cancer based on the description of the virtual support group. V007 noted a feeling of disconnect when the facilitator shared about her cancer experiences and could not relate to larger themes and experiences around loss and impact on self-esteem related to breast cancer, stating:

“*I was surprised that they didn’t have somebody who had breast cancer that was leading the group, I think that would’ve been more effective. Just somebody who could relate because … especially for women, you’re talking about effecting your self-esteem, you know, you’re taking your body parts away, what defines you as a woman essentially… when [the facilitator] would interject and talk about her [non-breast] cancer stuff, I’m like ‘it’s not the same’… you can’t really empathize when you’re dealing with a completely different situation in [other forms of] cancer, you don’t have your boobs cut off, you know? … You’re going through so many different things with breast cancer, so I really think that was a key thing*.”

Given this, V007 emphasized that when it comes to breast cancer, it is more important that the facilitator be knowledgeable about breast cancer than be a fellow female veteran, adding, “I value more somebody who can empathize with the pain that you’re going through… rather than that veteran aspect… ‘cause it’s a hard one for women.”

### 3.3. Virtual Aspect

Overall, veteran participants found the virtual format to be convenient and an effective platform for participation, with some noting that being able to speak with people far away and removed from their immediate lives was helpful. In addition, participants who lived in rural areas viewed joining the group as an opportunity to gain additional support. Veteran participant V001 explained, “I live out kind of in the middle of nowhere. And having a (virtual) support group, because of what I’m going through, I figured would be beneficial to me, to have contact with other people in similar situations. I don’t have anybody near me that’s going through what I’m going through.” However, privacy concerns were noted by some group members, specifically the potential lack of privacy from joining the virtual group from home. For example, V005 described, “One of the cons is that I need a space that is away from my husband where I can speak freely, and if he’s in the other room he can hear everything I’m saying.” Similarly, V009 shared, “Like my daughter would, when I was sitting there, want to come over and say ‘hi’. No, this is not a conversation for you to be involved in. [I was] struggling to create that separation.”

In addition, while veteran participants noted that meetings and appointments taking place virtually have become more common, some participants still described experiencing Wi-Fi connectivity issues or challenges accessing the telehealth platform. For example, while V010 joined a couple sessions by video, ultimately, she opted to join most sessions by telephone while driving home from work because her Wi-Fi connection was spotty, making the telephone a better way for her to participate. In contrast, V005 described how having some participants on video and others on the telephone was not ideal in her view, stating:

“*There are people who called in… I’m not saying that’s a bad thing if that’s all they can do, but that was a little distracting if you can’t see these people. There’s a value in facial expressions and we were laughing about different things. They don’t get that, and I never got to see some of these ladies*.”

Despite these challenges, HSL participant H003 viewed the benefits of being able to hold a national group as outweighing the limitations of the virtual platform. In contrast, while having access to a virtual group was particularly helpful for veterans who live in rural areas, some veteran participants expressed that they still would have preferred an in-person group. V008 weighed the pros and cons of participating in a support group virtually or in-person, stating:

“*I live far enough away from a [VA] facility that I was grateful that we had the opportunity… I think I would have rather walked in and sat with people than to have done the phone call because it just feels so disembodied. But at the same time there was a little bit anonymity. I know that it was easier maybe for me to be really honest and I’d like to believe that I was not the only one who was sharing things or feelings that were painful or challenging or scary, and it was good to have this as an option.*”

### 3.4. Group Structure

Some veterans reported feeling apprehensive about joining the virtual support group as they were not sure what the structure of the group would be, who the other group members would be, and thought that more details about the focus of the group at the outset would have been helpful. For example, V011 noted that it was not until she attended her first session that she understood what the group would be focused on and emphasized that it would have been helpful to get more detailed information, such as example session topics. However, once V011 attended the first session, she stated, “I loved it, it really was just what I needed because I was able to hear other veterans’ experience with cancer and also [the PSS] because she shared her experiences as well. So, yes, I loved it from the beginning when we first met virtually.”

Veterans expressed appreciation for the check-in at the start of each session, prompting each participant to share updates and making sure there was time for each person to speak. Reflecting on the check-ins that each session began with, V008 stated, “I think that my impression was that everyone welcomed the opportunity to be heard.” V010 relatedly stated, “I feel like [the check-ins] gave me an opportunity to participate, or voice my concern, or whatever else I had going on during that session, otherwise I would just sit in the back a lot of the time and be quiet.”

Feedback from veterans was mixed on whether group members preferred a rolling drop-in group versus a cohort-based model with a set number of weekly sessions. Several veteran participants liked the cohort-based model, arguing that this format can help build camaraderie among participants and that 6 weeks felt like too short of a time. In contrast, some participants argued that if the group was a rolling drop-in group instead of one with a set number of sessions, people could have the opportunity to join at any time versus waiting for the next cycle to begin. As V007 described, “I think it would be helpful for people who just got the diagnosis to not to have to wait … a certain time period to join.” V005 added that in a format with a set number of sessions, participants are also at risk of losing the support they had gained in the group once the group cycle ends. HSL participant H003 similarly heard feedback from veterans that for many, once their group cycle ended the community disappeared, and it was challenging for some to build a sense of community and then lose it, stating, “There was no continuation of it, the collaboration and communication between survivors kind of stopped essentially at the end of the six weeks. Cancer’s longer than six weeks. So it is kind of hard for those veterans to build a sense of community and then lose it.”

Many veteran participants were actively in treatment and could not attend all sessions in their series but expressed interest in having the option to drop-in to a group session whenever they were able to. If future offerings of the virtual peer support group remained for a set number of weeks, some veteran participants thought it would be helpful to have a more structured curriculum that focused on a dedicated topic or theme each week, including the use of handouts or discussion prompts. V005 commented that while the more open-ended structure of the group had benefits, it also at times placed the burden of what to discuss or do next on participants, stating,

“*It [group structure] was lacking in my opinion because there was no… curriculum, like today’s topic is X… I felt there could have been, ‘Let’s talk about family matters. How’s everyone coping with their family? Or your actual experience with your particular VA. Are you getting the support you need at your clinic*?”

Adding to the desire for a more structured curriculum, several veteran participants suggested the possibility of inviting guest speakers (e.g., a pharmacist) who could share specific expertise, new research, or new protocols coming out for breast cancer during sessions. Ultimately, the ideal described by veteran participants was to have multiple, simultaneous, virtual peer support group options—one set series with designated topics and one rolling drop-in group—and offer these groups at different times to accommodate more time zones and personal schedules. One HSL participant added that ideally there would be more than one PSS facilitator who could conduct groups in different time zones but noted that this is a challenge of being part of a national program.

### 3.5. Beneficial Topics

Session topics that veterans reported finding most helpful were topics that included discussion and sharing of resources (e.g., wigs). For example, veteran participant V004 highlighted that discussion and resources about how to discuss her diagnosis with her children were especially helpful. Several participants suggested that sharing resources should be centered as a focus of the group, rather than as an afterthought or bonus. Veteran participants also valued being assigned wellness-focused activities that they would report back on from week to week. For example, one assignment described was trying to plant something and sharing progress pictures from week to week of the plant growing. Another assignment focused on healthy eating, with prompts to share recipes and updates about what new healthy foods they tried. V011 described the benefit of these activities, stating, “It was to keep us busy and keep us focused on something positive, and I really enjoyed that.” When asked about whether these activities impacted her daily life, V011 explained:

“*It calmed down my depression and my anxiety, ‘cause I was constantly working on trying to make sure I had this project done so whenever we met again I could present it, ‘cause otherwise if I didn’t have that project all I would’ve been doing is just sitting around, taking medication, falling asleep, being depressed, so, it gave me an uplift*.”

Other veteran participants similarly shared that encouragement from the group to walk more throughout the day, exercise, and try new foods and recipes impacted their daily lives in a positive way. HSL participant H002 similarly heard feedback from veterans that they liked that the PSS did not only focus on cancer, but also encouraged discussions around wellness activities, stating:

“*It was a lot of, like, exchanging recipes and talking about life outside of cancer and strategizing how to get past [not] wanting to exercise or go for a walk. But they have side effects from their treatment and so, that was like one of the best feedback that I got from everybody was that they really liked that it wasn’t just about their cancer, it was about life and overcoming other obstacles*.”

At the same time, some participants expressed the view that the sessions were not well-balanced with regard to education and light-hearted content. While veteran participant V009 appreciated the intent to discuss lighthearted topics such as recipes and gardening, she emphasized that part of each session should focus on discussing breast cancer. V001 similarly shared a desire to spend more time in the group discussing experiences related to cancer (e.g., treatment, after-effects, feelings), rather than focusing on wellness activities, stating:

“*I would like to see more discussion on the issues we’re having. The group seems more focused on avoiding talking about that [and] talking about everything else, and so, we’re not discussing how we feel about what we’re going through and all that, it’s been more other topics to take our mind off of it more than dealing with the actual issues*.”

Other topics veteran participants wished had been covered during the peer support group included sexual wellness and feelings around loss of hair/eyebrows/eyelashes from cancer. V010 elaborated:

“*I had an esthetician put permanent eyebrows on me because my hair never really kind of came back after chemo and radiation, and to me I feel like that’s one of the better things that I’ve chosen to do for myself… I don’t know how many women feel the same way, but after 5 years of not having eyebrows and trying to draw them on, trying to stencil them in, trying to put little temporary eyebrows on, it’s like, ok, at least [now] I feel like more of a regular person with a face*.”

Despite varying experiences with the virtual peer support group, most participants stated that they would recommend the group to a friend.

### 3.6. Desired Outcomes by Health System Leaders

HSL participant H003 stated viewing the virtual peer support group as filling a gap in terms of applying a “whole health” approach to cancer care by providing veterans with support and community around a difficult diagnosis. He added that this pilot peer support group is unique because currently there is nothing else like it in VHA’s national tele-oncology program and not every VHA facility offers peer support programs. H003 continued:

“*I think the VA overall provides really good oncology care to patients, but one of the things the VA traditionally hasn’t invested in is like the ‘whole health’ of the veteran and really what I mean by that is like peer support, like the mental health that comes with getting like that difficult diagnosis… how do you navigate it, and I really think that the peer support really fills that gap for veterans, that there’s a community of veterans and there’s a group that they can come to share their experiences both navigating the VA, navigating family… so I think that’s a really critical mental health role*.”

When asked about what patient outcomes they might hope to see as a result of the pilot virtual peer support group, HSL participant H001 offered an interest in seeing improvement in quality of life metrics, reductions in cancer-related anxiety, and reductions in depression among group members, hopefully contributing to improvements in their cancer treatment experience. She stated:

“*I think there’s really good data that patients who are less anxious and less depressed tend to have better cancer outcomes. And it’s not because, like, their anxiety or their depression is making their cancer worse. It’s really just that they’re, you know, more likely to just go to their appointments and you know, not have chemotherapy delays or radiation delays and things like that*.”

H001 reasoned that a patient who has more community support or who is less anxious or depressed might potentially be more engaged in treatment. In contrast, H003 noted that the question of “outcomes” is a difficult question when it comes to peer-led programs, i.e., programs that are not delivered by a clinician. He argued that perhaps the outcomes will not necessarily be clinical outcomes when it comes to social support provided by peers. He elaborated:

“*It’s a difficult question. There’s really not gonna be clinical outcomes… they’re not gonna cure like a patient’s depression or something like that. The outcomes that I would expect to see aren’t necessarily empirical, but it’s just the general feeling from patients that there’s a community of veterans that are going through the exact same thing… So knowing that you have a community that you can connect with, shared in service and cancer… And that they feel connected that that they’re able to communicate about their diagnosis openly, honestly… and that they feel connected to a sense of community, I guess would be the outcome*.”

## 4. Discussion

This qualitative formative evaluation illuminated perspectives and attitudes about the acceptability, feasibility, and value of a newly formed national PSS-facilitated virtual peer support group for veterans living with breast cancer. We gained valuable data regarding strategies for support group recruitment, enrollment, and structure. Veteran participants noted the value of shared experiences, a need for the facilitator to be a peer with an overlapping shared lived experience, acceptability of the group being virtual, and uncovered attitudes and preferences regarding group structure, beneficial topics, and desired outcomes. Of note, more recent studies outside of the VHA in desperate populations have uncovered similar benefits when evaluating virtual peer support groups. Notably, one study found that patients involved in a virtual peer support group for other parents of children with eating disorders also appreciated that the groups provided connection to those going through a shared experience, social support, and decreased feelings of isolation [23]. Similarly, another noted that virtual support groups for those living with Parkinson disease effectively reduces social isolation and loneliness, and virtual groups ameliorate geographic isolation and are convenient [24]. HSLs noted that target outcomes might be linked to feeling connected with community and having an increased feeling of support, which in turn may affect clinical outcomes (e.g., anxiety, depression).

From a feasibility perspective, this pilot demonstrated that a national virtual peer support group for women veterans with breast cancer could be successfully recruited, delivered, and staffed within existing VHA infrastructure. Recruitment through the BGSOE Nurse Navigator and VetConnect dashboard enabled identification and outreach to eligible veterans across sites, resulting in delivery of seven six-week waves with sustained participation. Sessions were staffed by an existing PSS whose facilitation and documentation responsibilities were integrated into her routine role, supporting staffing feasibility without additional personnel. Use of the VVC platform enabled national delivery, with connectivity challenges noted by some participants but not preventing group participation.

Because most VHA Oncologists do not see a high enough volume of cases to provide in-house surgical services for breast cancer, care is typically provided in the community which can lead to a feeling of isolation, psychosocial distress, and frustrations around care coordination [2,3,4,5,6,7]. By creating centralized in-house subject matter expertise to guide oncologic care for breast cancer, BGOSE’s aim is to decrease utilization of community care and ensure veterans being treated for these cancers do not lose touch with their VA communities. Furthermore, by having a national footprint, BGSOE can provide robust resources to small pockets of geographically disbursed veterans. Our findings provide initial evidence of the need for and importance of building community and support for women veterans with breast cancer through peer-delivered support groups. We suggest that for organizations adopting virtual peer support groups across large geographic areas, offering multiple group formats throughout multiple times per week is important to accommodate a wider group of participants.

**Figure 3:**
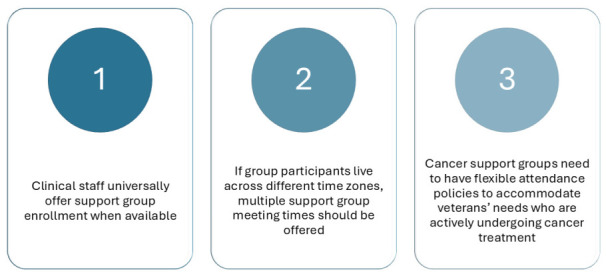
Key takeaways from a formative evaluation of a demonstration pilot of a peer support-facilitated virtual support group for women veterans with breast cancer.

Related research has shown that support groups have long suffered recruitment barriers and peer groups face high attrition [18]. There are several factors that influence those challenges, including that providers and patients are often unaware that such groups exist and that patients may have mismatched expectations regarding the services [25]. We expand on this prior research and found that, while group delivery over a national catchment was helpful in reducing recruitment barriers, attrition still occurred when the group did not align with personal schedules (e.g., cancer-related medical appointments). We also found that recruitment challenges were eased when the BGSOE Nurse Navigator used scripted language and universally offered enrollment into the support group as part of her standard intake protocol. Based on our findings, we suggest: (1) clinical staff universally offer support group enrollment when available; (2) if group participants live across multiple time zones, multiple support group meeting times should be offered; (3) cancer support groups need to have flexible attendance policies to accommodate veterans’ needs who are actively undergoing cancer treatment (Figure 3).

We found that veteran participants greatly valued being able to share experiences and connect with other women veterans with breast cancer. Veterans described benefits from peer interactions including supportive coping as well as instrumental coping. Additionally, veterans noted the strength of the wellness topics but desired more balance with cancer-related topics and sharing available resources. They also expressed a desire for the PSS to have a personal experience with breast cancer to amplify the supportive effects. Of note, none of the interview participants expressed harm or negative impacts resulting from the group dynamics. These findings highlight the fact that cancer support groups, unlike more traditional mental health support groups typically found at the VHA, do require a degree of specificity regarding programmatic content and PSS-cancer-experience-matching for group experience to feel authentic and meaningful [15]. As VHA programs outside of mental health, and non-VHA health care systems consider offering PSS services to patients, the question of “who is a peer” will need to be deeply considered. Limited resources and budgets impact recruiting and retaining a PSS with a matched background, which may be near impossible for smaller healthcare systems, and the virtually delivered nature and national catchment of the group can help with such feasibility. Our work suggests in the specific case of women veterans with breast cancer, that matching on gender, veteran-identity, and a prior cancer diagnosis is not sufficient, and the PSS matching requires the additional note of specific cancer-type concordance.

While this work has strengths, including potential transferability of findings given utilization of a national cohort of participants, there are also limitations. Our sample size was relatively small, and our findings may be influenced by self-selection bias (i.e., participants who agreed to an interview may have had a more positive assessment). We did not survey or interview veterans who declined participation in the support group. Additionally, our pilot aimed to determine if we could successfully recruit veterans with breast cancer to participate in a virtual support group and if veterans would attend such groups. While it was outside our scope to make any inferences about the clinical impact of the support groups, based on participation in the support group waves and qualitative data, we posit that the support groups were utilized and that participants and clinic leadership found merit in the experience. In addition, all study authors contributed to interpreting findings and the implications of the study. However, it is likely that the primary qualitative team members’ identities as women and professional backgrounds as women’s health researchers within VHA influenced interpretations of the data. To avoid speaking for the data, the qualitative team made efforts to discuss and validate emerging findings with the larger study team as well as pairing findings with direct participant quotations to ensure alignment with participants’ voices.

Future work should include a larger summative study including a control group and a continued effort should be made on recruitment and retention of rural veterans. Additional qualitative interviews including interviews with veterans who decline participation in the support group, validated survey tools to measure the impact of the intervention (e.g., support, isolation, wellbeing), and fidelity to treatment protocols (such as continuation of tamoxifen) would help our understanding of outcomes. In addition, a formal cost-effectiveness analysis will provide valuable information needed to implement and disseminate virtual peer support groups more broadly. Considering we found conflicting preferences for a cohort-based group vs. drop-in group and for a focus on wellness vs. cancer-related topics, it would be helpful to understand if these preferences are driven by phase of treatment and immediate needs.

## 5. Conclusions

This formative evaluation highlights the potential public health value of virtual peer support programs as a scalable approach to addressing psychosocial needs among women veterans with breast cancer, a geographically dispersed and clinically vulnerable population. Findings suggest several practical implications for implementation of virtual peer support programs in oncology settings. Recruitment may be strengthened by embedding universal offers of support group enrollment within routine nurse navigator intake workflows. Group format should remain flexible, with both cohort-based and rolling drop-in options offered across multiple time slots to accommodate treatment schedules and geographic dispersion. Staffing models should prioritize peer support specialists with cancer-type/concordant lived experience when feasible; when this is not possible, programs should set clear expectations and supplement peer facilitation with targeted educational resources or guest clinical experts. The BGSOE virtual breast cancer support group is now in its second year with a new peer support specialist who is a breast cancer survivor and is piloting additional formats, including drop-in hours and a virtual resource library. This pilot formative evaluation provided critical data to inform recruitment, structure, and content of peer-facilitated cancer support groups and underscores the potential role of virtual peer support in reducing social isolation, strengthening community, and supporting wellbeing among patients receiving cancer care across fragmented systems. Future work should incorporate validated psychosocial measures and comparative designs to better assess impacts on wellbeing, social support, and treatment engagement.

## Figures and Tables

**Table 1:** Participant demographic characteristics.

	Participants (*n* = 41)	Qualitattive Interviewees (*n* = 11)
Age		
30–39	7% (*n* = 3)	18% (*n* = 2)
40–49	10% (*n* = 4)	18% (*n* = 2)
50–59	37% (*n* = 15)	27% (*n* = 3)
60–69	29% (*n* = 12)	27% (*n* = 3)
70–79	15% (*n* = 6)	0
80+	2% (*n* = 1)	9% (*n* = 1)
Race		
White	32% (*n* = 13)	54% (*n* = 6)
Black	54% (*n* = 22)	27% (*n* = 3)
American Indian or Alaska Native	0	0
Asian	2% (*n* = 1)	0
Native Hawaiian or Other Pacific Islander	2% (*n* = 1)	9% (*n* = 1)
Decline	2% (*n* = 1)	0
Unasnwered	5% (*n* = 2)	0
Miltiracial	2% (*n* = 1)	9% (*n* = 1)
Ethnicity		
Not Hispanic/Latino	90% (*n* = 37)	91% (*n* = 10)
Hispanic/Latino	2% (*n* = 1)	0
Decline	2% (*n* = 1)	0
Unasnwered	0	0
Unknown	5% (*n* = 2)	9% (*n* = 1)
RUCA* Score		
1	83% (*n* = 34)	82% (*n* = 9)
1.1	2% (*n* = 1)	9% (*n* = 1)
2	7% (*n* = 3)	0
4	2% (*n* = 1)	0
10	5% (*n* = 2)	9% (*n* = 1)

* RUCA: Rural-Urban Commuting Areas.

## Data Availability

Data supporting reported results are available and can be accessed by contacting the corresponding author.

## Supplementary Materials

The following supporting information can be downloaded at https://www.mdpi.com/article/10.3390/ijerph23060817/s1, S1: Veteran interview guide; S2: Health System Leader interview guide.
