# Supplementary material for: A National Virtual Peer Support Group for Women Veterans Living with Breast Cancer: Lessons from the Field
Source: Int J Environ Res Public Health. 2026 Jun 19;23(6):817. doi: 10.3390/ijerph23060817 (PMC13299964; doi:10.3390/ijerph23060817)
Supplement: Supplementary file 1 [file ijerph-23-00817-s001.zip › ijerph-4260599-supplementary.pdf]

<u>Supplementary Materials</u>

S1. Veteran interview guide

Interview Guide. Thanks for talking with me today. As you know, I am a health researcher at the VA Central Western Massachusetts Healthcare System/ San Francisco VA Health Care System. I am conducting this interview to learn more about your virtual peer support group experience. As a reminder, your participation in this interview is completely voluntary, and you can choose not to participate or answer any of the questions I ask you. We will use the information we gather to understand how to improve cancer support groups for women Veterans through changes to the program, reports and manuscripts, but we will never include any personally identifiable information. The data we collect today will be stored securely and only accessed by approved study staff. Are you still interested in participating? *If yes, go to the next item.*

- Do you consent to this interview being audio recorded?
- Do you have any questions before we get started?

1. To start, I'd like you to think back to when you were invited to participate in the virtual peer support group. What were your initial thoughts about joining a virtual peer support group?
    a. Probe: What made you interested in joining this group?
    b. Probe: What concerns did you have?
    c. Probe: Were there any aspects you were most looking forward to? Have you participated in any other type of support group in the past? If so, how did this experience differ?
2. How many of 6 session cycles have you participated in?
3. What are your views on the virtual aspect of participation?
    a. Probe: Did you have any trouble connecting to the virtual group?
    b. Probe: How did you feel about the quality of the connection?
    c. Probe: Do you feel that you were able to participate effectively through a virtual platform?
4. How did you feel about the overall structure of the support group?
    a. Probe: What were your favorite aspects of the group?
    b. Probe: What were you least favorite aspects of the group?
    c. Probe: Did you feel that the moderator was effective in keeping the discussion going?
5. How did you feel about the number of sessions (six)?
    a. Probe: Would you have liked to meet more or less often, why or why not?
6. [Describe the structure of each session (e.g., a check-in, structured activity).] What are your views on the session structure?

7. [Describe some of the session topics that are covered from week-to-week.] What are your perspectives/views on the session topics?
    a. Probe: Were session topics useful/helpful, why or why not?
    b. Probe: Were there any topics that were unhelpful or didn't appeal to you, why or why not?
    c. Probe: Was there anything that you wished you could have discussed that was not brought up during the peer support group?
8. How comfortable were you sharing your personal experiences in the peer support group? Why or why not?
9. Do you have any suggestions for improvement?
    a. Probe: Tell me more…/Ask for specific examples
10. Would you recommend the virtual support group to a friend? Why or why not?
11. How did the topics discussed in the support group influence you in your daily life?
    a. Probes: Tell me more about that/Can you give me a specific example?
12. Are you considering participating in any future cycles of the cancer support group? Why or why not?

S2. Health System Leader interview guide

- **The purpose of our interview today is…**
- **Confirm time availability – 30 minutes**
- *OBTAIN VERBAL CONSENT*
    - **Voluntary**; participants can
        - Opt-out any time, no questions asked
        - Decline to answer questions
        - End the interview
    - **Confidentiality**
        - No one at facility will be notified about participation
        - Anything said will be kept in confidence
    - **Privacy**
        - Responses will be kept anonymous
        - Responses will be de-identified and assigned a participant ID number
- ***Begin MS Teams transcription, confirm consent***

I. Professional background

1. Please tell me a little about your professional background.
    a. (clinical, business, or other degree)
2. What is your role at the VA? (title, brief description)

    a. How long have you been in your current role at VA?
    b. How many years have you been in practice in total?
3. Can you describe to me your connection to the NTO/BGSOE/[PSS-led virtual breast cancer support group]?
    a. Probe: Role in recruitment or referral processes to the group

II. Clinic Context

4. Can you describe the patient population cared for within VA NTO/BGSOE?
    a. Probe: (Clinician) Describe the patient population you see in your own clinic.
    b. Probe: Views on patient accessibility to care/services
5. What are some of the strengths of NTO/BGSEO?
6. What are some of the challenges of NTO/BGSOE?

III. Appropriateness

7. In your, what is the objective of the [PSS-led virtual breast cancer support group]?
    a. Probes: Current gaps in services or resources
8. In what ways might the [PSS-led virtual breast cancer support group] help meet patient needs?
9. In what ways might the [PSS-led virtual breast cancer support group] help meet provider needs?
10. Are there any kinds of patient outcomes from the [PSS-led virtual breast cancer support group] that you would hope to see?
11. (Clinician) What are some factors that might prompt you to refer a Veteran you're caring for to the [PSS-led virtual breast cancer support group]?

IV. Acceptability

12. What feedback have you heard from Veterans who have participated in the [PSS-led virtual breast cancer support group] about their experience?
13. What kinds of evidence about PSS-led programming would encourage wider adoption of the [PSS-led virtual breast cancer support group]?

Feasibility

14. What kinds of changes or adaptations are needed to make the [PSS-led virtual breast cancer support group] work well in NTO/BGSOE/in your clinic?
    a. How feasible are these changes?
15. Do you have any general suggestions for improving the [PSS-led virtual breast cancer support group]?

V. Sustainability

16. Are there any kinds of policies or guidelines would support wider use of the [PSS-led virtual breast cancer support group]?
17. What factors might make it challenging to growing the [PSS-led virtual breast cancer support group]?
18. What factors can support the [PSS-led virtual breast cancer support group]'s sustainability long-term?
